# Evaluation of a droplet digital PCR assay for quantification of *Mycobacterium avium* subsp. *paratuberculosis* DNA in whole-blood and fecal samples from MAP-infected Holstein cattle

**DOI:** 10.3389/fvets.2022.944189

**Published:** 2022-09-30

**Authors:** Gerard Badia-Bringué, Maria Canive, Rosa Casais, Cristina Blanco-Vázquez, Javier Amado, Natalia Iglesias, Aitor González, Mertxe Bascones, Ramon A. Juste, Marta Alonso-Hearn

**Affiliations:** ^1^Department of Animal Health, NEIKER- Basque Institute for Agricultural Research and Development, Basque Research and Technology Alliance, Derio, Spain; ^2^Doctoral Program in Molecular Biology and Biomedicine, Universidad del País Vasco/Euskal Herriko Unibertsitatea (UPV/EHU), Leioa, Spain; ^3^Center for Animal Biotechnology, Servicio Regional de Investigación y Desarrollo Agroalimentario, Deva, Spain; ^4^Department of Microbiology, Laboratorio Regional de Sanidad Animal del Principado de Asturias, Gijón, Spain; ^5^Department of Conservation of Natural Resources, NEIKER- Basque Institute for Agricultural Research and Development, Basque Research and Technology Alliance, Derio, Spain

**Keywords:** droplet digital PCR, *paratuberculosis*, molecular diagnosis, blood, feces

## Abstract

Bovine paratuberculosis (PTB) is an infectious disease that affects ruminants worldwide and is a burden on the dairy industry. PTB control measures include culling of *Mycobacterium avium* subsp. *paratuberculosis* (MAP)-infected animals from the herd and the enhancement of farm-biosecurity measures. Diagnostics tools for the direct detection of MAP are fecal real-time qPCR and bacteriological culture, the last one being considered the gold standard. However, both show limitations for detecting subclinical MAP-infected cattle with low bacterial load in feces and gut tissues. Droplet digital polymerase chain reaction (ddPCR) is a third-generation PCR method that shows high reproducibility for the quantification of low DNA copy numbers. The objective of this study was to design a ddPCR assay to detect and quantify a fragment of the F57 MAP-specific sequence in samples of naturally MAP-infected Holstein cattle. DNA was isolated from whole-blood and fecal samples from control cows with a negative ELISA and qPCR result (*N* = 75) and from cows with PTB-associated focal (*N* = 32), multifocal (*N* = 21), and diffuse lesions (*N* = 17) in gut tissues. After ddPCR, the DNA extracted from fecal samples of cows with diffuse lesions showed higher mean copies per microliter (13,791.2 copies/μl) than samples from cows with multifocal lesions (78.8 copies/μl), focal lesions (177.1 copies/μl) or control cows (4.8 copies/μl) (*P* ≤ 0.05). Significant differences in mean DNA copies/μl were also observed in the blood samples from cows with focal lesions (47.7 copies/μl) when compared with cows with multifocal and diffuse lesions; 18.1 and 12.4 copies/μl, respectively. Using a principal component analysis, the results of the fecal ddPCR clustered together with the results of a commercial ELISA for the specific detection of MAP antibodies, fecal and tissue qPCR, and bacteriological culture results. In contrast, blood ddPCR results clustered together with the results of an ELISA for the detection of a biomarker of subclinical PTB, the ABCA13 transporter. Blood ddPCR was the most sensitive tool (sensitivity 71%, specificity 100%) of all the quantitative methods used in the study for the detection of subclinical cows with focal lesions.

## Introduction

Bovine paratuberculosis (PTB) or Johne's disease is caused by *Mycobacterium avium* subsp. *paratuberculosis* (MAP), and is a widespread infectious disease that negatively impacts both the dairy industry and animal welfare. Several studies have demonstrated that more than 50% of the dairy cattle herds are positive for MAP antibodies in the USA and in Europe, which makes bovine PTB endemic in these areas (1, 2). Estimates show global economic losses exceeding US1.5 billion dollars annually from PTB cases, with US198.42 million dollars in the United States and US364.31 million dollars in Europe (3). Infection usually occurs at an early stage of life, and in some cows, it can remain subclinical for years. After being ingested, MAP crosses enterocytes and M cells of the intestinal Peyer's patches and it is phagocytized by sub-epithelial macrophages and dendritic cells. MAP can survive within infected macrophages by inhibiting apoptosis and phagosomal acidification, and by preventing the presentation of antigens to the immune system (4). Infected macrophages dissemination *via* the lymphatic or blood system results in the development of PTB-associated lesions in susceptible animals (5). Clinical signs usually occur >24 months of age (6) but only 10–15% of the infected cattle develop clinical signs including progressive weight loss, diarrhea, and decreased milk yield (7). As the infection progresses, the lesions in the intestine and lymph nodes become more abundant and the granulomatous infiltrate becomes diffuse disrupting the mucosal structure and affecting jejunum and ileum (8, 9). Whitlock et al. (10) described three stages of infection with MAP: (i) in the first stage, cattle do not shed MAP in the feces and do not exhibit clinical signs, (ii) in the second stage, cattle shed intermittently MAP in the feces but do not exhibit clinical signs, and in (iii) in the third stage, cattle shed MAP in the feces and exhibit clinical signs (10). MAP infection has been associated with the inflammatory bowel disease, several autoimmune diseases, and colorectal cancer in humans (11, 12).

Nowadays, MAP control strategies are focused on the improvement of hygiene measures and the diagnosis and elimination of infected animals. The most common diagnostic tests used to detect MAP are ELISA for the detection of MAP antibodies and real-time PCR for the detection of MAP DNA in fecal samples. However, they present several limitations. Although serum ELISA is a simple, fast, and cost-effective method for PTB diagnosis, it is known to have low sensitivity for MAP-infected animals that do not show clinical signs. The sensitivity of serum-specific antibody ELISA varies, being 50–87% in cattle with clinical signs, 24–94% in cattle with no clinical signs but shedding MAP, and 7–22% in infected cattle with no clinical signs and no shedding (13). On the other hand, fecal real-time PCR suffers from limitations such as inhibition problems due to the presence of PCR inhibitors in fecal samples and low sensitivity for detection of subclinical cattle (14). Presently, fecal bacteriological culture is regarded as “the gold standard” test for the ante-mortem diagnosis of MAP infection (15). However, bacteriological culture from feces is a slow procedure since MAP generation time is higher than 24 h and its sensitivity has been reported between 39.0 and 92.0%, depending on the stage of infection of the tested animals (16, 17). Therefore, the detection of subclinical infections remains a challenge, and the development of more sensitive tests to detect subclinical MAP-infected cattle are crucial.

Droplet digital polymerase chain reaction (ddPCR) is an emerging technology for nucleic acid detection and absolute quantification based on DNA amplification and water-oil emulsion droplet technology (18). This technology partitions the sample into thousands of single-nanoliter-sized droplets, so that each droplet may contain one or multiple copies of the target DNA or none. Then, a standard PCR is performed, and a droplet reader labels each droplet as positive or negative depending on whether it contains the amplified target DNA or not by reading their fluorescence. Finally, absolute DNA concentration is measured by calculating the negative to positive droplets using Poisson statistics, without the need for a calibration curve which simplifies the quantification and reduced results variability and cost (19). ddPCR has been proved to be less sensitive to inhibitors than qPCR due to sample partitioning and more accurate in absolute quantification of low-copy nucleic acids than qPCR. ddPCR has already been used for the detection of low amounts of pathogens including *Mycobacterium tuberculosis* (20–23), and is a robust technique to quantify MAP genomic DNA (24, 25). However, ddPCR capabilities as a MAP detection tool in clinical samples have not been tested yet.

In this study, we designed a ddPCR assay to effectively detect and quantify a 77 bp fragment from the one copy F57 MAP-specific gene. We assessed the capacity of ddPCR for the detection of MAP in feces of naturally infected cattle with PTB-associated focal, multifocal, and diffuse lesions in intestinal and lymphoid tissues. In addition, the potential of ddPCR for detecting circulating MAP was evaluated in blood samples. As controls, we used cattle from a PTB-free farm with negative ELISA results in three consecutive years, and control cattle without histopathological lesions and with negative ELISA and fecal and tissue qPCR and bacteriological culture at slaughter. The results of ddPCR were compared with those obtained with other diagnostic procedures such as ELISA for MAP antibodies, fecal and tissue qPCR, fecal and tissue bacteriological culture, and an ELISA for the detection of the ATP-binding cassette subfamily A member 13 (ABCA13). This ABCA13 ELISA was previously validated for the detection of cows with focal lesions, the most frequent lesion in the subclinical stages of the disease (26, 27). To our knowledge, this is the first report on the use of ddPCR for the detection of MAP in blood and fecal samples from cattle with distinct PTB-associated lesions in gut tissues.

## Materials and methods

### Ethical statement

All experimental procedures performed on the animals in this study were approved by the Animal Ethics Committee of the Servicio Regional de Investigación y Desarrollo Agroalimentario (SERIDA) and authorized by the Consejería de Agroganadería y Recursos Autóctonos of the Principality of Asturias (approval code PROAE 29/2015 and PROAE 6672019). All the procedures were carried out following the European Guidelines for the Care and Use of Animals for Research Purposes (2012/63/EU). Blood, gut tissues, and fecal samples were collected by trained personnel and in accordance with good veterinary practices.

### Animals and PTB infectious status

The cows included in this study (*N* = 145) came from two different populations. The PTB-free group consisted of 71 Holstein cows from a PTB-free farm in Asturias, Spain (Supplementary Table 1). The PTB-free status of this farm was verified each year from 2016 to 2019 by ELISA to detect the presence of anti-MAP antibodies in serum samples using the *Mycobacterium paratuberculosis* Antibody test (IDEXX laboratories, Hoofddrop, the Netherlands). In 2019, fecal qPCR using DNA isolated from fecal samples of all the animals in the herd was performed to confirm the PTB-free status of the farm (26). The slaughtered cows included in the study consisted of 74 cows from a commercial dairy farm in Asturias with a mean prevalence of PTB of 6.30 % based on ELISA results (IDEXX laboratories). The PTB infectious status of these 74 cows at the time of slaughter was also determined by histopathological analysis, and bacteriological culture and qPCR of gut tissues and feces as previously described (26, 28). While samples of feces were collected from all the animals for fecal ddPCR, blood samples were only taken from the slaughtered animals for blood ddPCR.

For ELISA, blood was collected for the coccygeal vein of each animal into 4.5 ml serum clot activator Vacutainer® tubes (Vacuette, Kremsmunster, Austria) and centrifuged at 2,500 × g for 20 min to allow serum separation. The IDEXX ELISA was performed according to the manufacturer's instructions and the optical density (OD) of each sample was measured at 450 nm in an ELISA plate reader (model 680, Sigma, St Louis, MO). Final OD values were normalized and the results were expressed as the percentage of the positive control OD according to the equation:


$$\begin{array}{l} ELISA\left(\%\right)=\frac{\begin{array}{c} {OD\left(sample\right)}_{+Ag}\\ -{OD\left(sample\right)}_{-Ag} \end{array}}{\begin{array}{c} OD{\left(mean\;positive\;controls\right)}_{+Ag}\\ -{OD\left(mean\;positive\;controls\right)}_{-Ag} \end{array}}\;\times \;100 \end{array}$$

The concentration (pg/ml) of the ABCA13 biomarker in the serum of the animals was measured using a commercially available ELISA (MyBiosource, San Diego, CA, USA) as previously described (26).

For histopathological analysis, samples from the distal jejunum, ileocecal valve (ICV), and jejunal and ileal lymph nodes were collected and fixed in 10% neutral buffered formalin, dehydrated through alcohol gradient, and embedded in paraffin wax using standard procedures. Samples were then cut into 4 μm sections using a microtome and stained with hematoxylin-eosin (HE) and Ziehl-Neelsen (ZN). The stained sections were examined by light microscopy to classify samples into four groups: no lesions, and with focal, multifocal, or diffuse lesions (8).

For tissue bacteriological culture, samples from ileocecal lymph nodes, distal jejunal lymph node, ICV, and distal jejunum were pooled (2 g), decontaminated with 38 ml of hexadecyl pyridium chloride (Sigma, St. Louis, MO, USA) at a final concentration of 0.75% and homogenized in a Stomacher blender. After 30 min of incubation at room temperature, 15 ml of the suspension were transferred to a new tube and left overnight for decontamination. The next day, 200 μl of the suspension were inoculated into two slants of Herrold's egg yolk medium (HEYM; Becton Dickinson, Sparks, MD) and two slants of Lowenstein-Jensen medium (LJ; Difco, Detroit, MI), both supplemented with 2 mg/L of Mycobactin J (Allied Monitor, Fayette, MO). For fecal bacteriological culture, feces (2 g) were taken from the rectum of each animal, homogenized, decontaminated, and cultured as described for tissue bacteriological culture. Bacterial load was classified as low (< 10 cfu; estimated average 2 cfu/tube), medium (between 10 and 50 cfu, estimated average 20 cfu/tube), or heavy (>50 cfu; estimated average 200 cfu/tube).

Isolation of genomic DNA from gut tissues and feces was performed using the MagMAX Total Nucleic Acid Isolation Kit according to the manufacturer's instructions (Thermo Fisher Scientific, Lissieu, France). At the end of the extraction procedure, the DNA was eluted in 90 μl of elution buffer. Aliquots of DNA samples were stored at −20°C until used in downstream PCR assays. Real-time quantitative PCR (qPCR) was conducted using the ParaTb Kuanti–VK kit (Vacunek, Bizkaia, Spain) in duplicate as previously described (29). The kit uses a F57 TaqMan probe labeled with the fluorescent reporter dye 5-carboxyfluorescein (FAM) at the 5′ end. Inhibition of the amplification reaction is ruled out by including an internal hybridization probe labeled with 6-carboxy-4′,5′-dichloro-2′,7′-dimethoxyfluorescein, succinimidyl ester (JOE) at the 5′ end. Real-time qPCR amplifications were performed on an ABI Prism 7500 detection system (Applied Biosystems, Carlsbad, CA) under the following conditions: 1 cycle of denaturation at 95°C for 10 min, 45 cycles of denaturation at 95°C for 15 s, and annealing/extension at 60°C for 60 s. Quantification of the MAP-specific F57 sequence DNA copy numbers in unknown samples was accomplished by duplicate using a standard curve generated with a series of known quantities of the target sequence ranging from 10^7^ to 10 copies and according to the manufacturer's instructions. The quantification results were divided by 5 (5 μl of DNA were loaded into each PCR mixture) and multiplied by the total volume used for elution (90 μl) to assess the number of F57 copies estimated for the whole volume of DNA extract. DNA extracts represent 0.175 μl of a mixture containing 1 g of feces or tissues resuspended in 3.33 ml of PBS. Thus, the number of copies calculated for DNA extracts was multiplied by the corresponding volume used to resuspend 1 g of feces or tissues and divided by the starting volume used. Final data were expressed as copies of MAP DNA per gram.

### DNA extraction from MAP bacterial culture and whole blood samples

To verify the correct performance of the ddPCR, the bovine K10 isolate of MAP was grown in T25 tissue culture flasks at 37 ± 1°C in 8 ml of Middlebrook 7 H9 broth (Difco Laboratories, Detroit, MI) supplemented with 10% (v/v) oleic acid-albumin-dextrose-catalase (Becton, Dickinson and Company, Franklin Lakes, NJ), 0.05% (v/v) Tween-80 (Sigma-Aldrich, St Louis, MO) and 2 mg/L of Mycobactin J (Allied Monitor Inc., Fayette, MO) for 30 days at 37°C. Bacterial cells (1 ml) were vortexed to mix the sample and heated at 95°C for 20 min. Whole-blood samples were collected from the coccygeal tail vein of the animals into EDTA Vacutainer tubes (BD Vacutainer system). DNA was extracted from whole blood samples and MAP bacterial culture using MagMAX Total Nucleic Acid Isolation Kit according to the manufacturer's instructions (Thermo Fisher Scientific, USA). The concentration of the purified DNAs was determined by measuring the UV absorbance at 260 nm in a Nanodrop ND-1000 Spectrophotometer (Thermo Fisher Scientific, USA).

### Primer design

MAP-specific F57 sequence was downloaded from the nucleotide database National Centre for Biotechnology Information (NCBI) (reference X70277.1), and appropriate primers were predicted using Primer3Plus (30). Criteria for single primers design included: primers should have a GC content of 40–60%, a melting temperature between 50 and 65°C, primers size should be no smaller than 13 and no larger than 30 nucleotides, they should not include repetitions of more than three consecutive G-C, and Gs and Cs should be the furthest 3′ nucleotides when possible. Because primers were used in pairs, we checked that paired primers sequences did not exhibit significant complementarity between 3′ ends because this can result in primer dimers which can decrease or prevent amplification. The presence of secondary structures in the region where the primers hybridize should be avoided, the chosen region should ideally have a GC content of 40–60%, and the amplification product size should be between 60 and 200 bp. To ensure the specificity of the primers to our target sequence, we used the Basic Local Alignment Search Tool (BLAST) hosted at the NCBI. We also checked for the secondary structure of the amplicon using the Mfold program (http://mfold.rna.albany.edu). The sequences of the specific primers used to amplify the MAP-specific F57 sequence were as follows: forward: 5′-AAC GCT TGG CAC TCG TCA ATC AC-3′ (Tm = 58.4°C) and reverse: 5'-TCG TCC AAC TTT TGG GAT CGC GG-3′ (Tm = 59.8°C). The amplification product was 77 bp in length. The oligonucleotides used as primers were synthesized by Invitrogen (Thermo Fisher Scientific, USA).

### HindIII digestion

Restriction digestion of the DNA samples outside the amplicon region improves the overall performance of ddPCR by making the template more accessible and reducing sample viscosity. We selected the HindIII enzyme (A/AGCTT) because it does not cut within the F57 PCR amplicon and it is insensitive to methylation and therefore avoids incomplete fragmentation due to methylation of the target DNA. Two strategies were used to perform restriction digestion of DNA samples: digestion directly in the ddPCR reaction or conventional digestion before ddPCR. For ddPCR of MAP DNA samples isolated from bacterial cultures, 1 μl of HindIII (5 U/μl) (Takara, Shiga, Japan) was added to the ddPCR reaction. For ddPCR of MAP DNA isolated from animal samples, HindIII digestion was carried out prior ddPCR as a separate reaction in a volume of 12 μl containing 9.8 μl of DNA, 1.2 μl HindIII buffer, and 1 μl HindIII (5 U/μl). Each reaction was incubated for 2 h at 37°C and the digested DNA was immediately used in a ddPCR experiment or stored at −20°C.

### ddPCR procedure

In the ddPCR assay, the reaction mix included 1x QX200™ ddPCR™ EvaGreen® Supermix (Bio-Rad, Hercules, CA, USA), 200 nM of F and R primers, target DNA, and DEPC-treated water in a final volume of 21 μl. For ddPCR amplifications of MAP DNA isolated from bacterial culture, 10 ng of DNA and 1 μl of HindIII (5 U/μl) were included in the ddPCR reaction mix. For ddPCR of animal samples, two ddPCR reactions containing 9 and 2 μl of HindIII-digested DNAs were prepared to ensure that one of them is within the optimal digital range. Each reaction mix (21 μl) was slowly dispensed into the bottom of the well of a DG8™ Cartridge (Bio-Rad, USA), making sure bubbles were not generated. Next, 70 μl of droplet generation oil for Eva Green® (Bio-Rad, USA) were loaded into the bottom of the oil wells of the DG8™ Cartridge, and the cartridge was covered with a DG8™ Gasket (Bio-rad, USA) and placed into the QX200 Droplet Generator (Bio-Rad, USA). The droplet generator partitions each sample into 15,000-20,000 nanoliter-sized droplets. After droplet generation, droplets were transferred to a 96-well plate (Bio-Rad, USA) with a RAININ p-50 pipette. The pipetting was performed slowly and gently so as not to shatter the droplets or generate bubbles. The PCR plate was heat sealed with a pierceable foil using the PX1™ PCR Plate Sealer (Bio-Rad, USA) at 180°C for 5 s. After heat sealing, the PCR plate was placed in a T100™ Thermal Cycler (Bio-Rad, USA) for PCR using the following cycling conditions: an initial denaturation cycle at 95°C for 30 s was followed by 40 cycles of 30 s at 95°C and 1 min at 65°C. A final signal stabilization cycle at 4°C for 5 min followed by 90°C at 5 min was performed. A 2°C/s ramp rate was used to ensure that each droplet reached the correct temperature for each step during the cycling. Good laboratory practices for PCR were followed (31).

### ddPCR data acquisition and analysis

Following PCR amplification, the PCR plate was placed in a QX200 Droplet Reader (Bio-Rad, USA), which counts the fluorescent positive and negative droplets in each sample. Then, *QuantaSoft*™ (version 1.7.4.0917) and *QuantaSoft*™ *Analysis Pro* (version 1.0.596) softwares (Bio-Rad, USA) were used for automatic thresholding and absolute quantification (in copies/μl) of the target sequence in the final 1x ddPCR reaction. Data from 12,000–16,000 droplets were used for concentration calculations. Samples with a low number of droplets (< 10,000) were excluded from the analysis. As suggested by the Bio-Rad ddPCR manual, samples were labeled as positive if they contained at least three positive droplets and the observed positive droplets in the negative controls were zero. If all partitions are of equal volume, the mean concentration of target molecules per partition (λ) can be estimated from the probability that a partition is negative using the proportion of negative partitions and the Poisson distribution (32). This concentration is derived from the number of positive partitions (κ) and the total number of partitions in the reaction (*n*):


$$\begin{array}{l} \lambda \;=\;-\ln\;\left(1\;-\;\frac{\kappa }{n}\right) \end{array}$$

We also accounted for the concentration of the template in the restriction reaction to calculate MAP concentration in the original DNA sample. The total number of copies per μl in the original samples was calculated as follows:


$$\begin{array}{ll} \frac{\text{copies}}{\text{μl}}\;\left(in\;original\;sample\right)\\ = & \;\frac{\left(\frac{\text{copies}}{\text{μl}}\;\left(in\;ddPCR\;reaction\right)\right)A\;B\;C\;}{\text{D E}} \end{array}$$

A*: Total volume of ddPCR reaction mix (*μ*l)*

B*: Dilution factor, if the sample was diluted*

C: *Total volume of the digestion reaction, if performed (*μ*l)*

D: *Total DNA loaded to the ddPCR reaction mix (*μ*l)*

E: *Total DNA loaded to the digestion reaction, if performed (*μ*l)*

The current manuscript addresses known requirements for ddPCR and the minimum information for publication of quantitative digital PCR experiments (32, 33).

### Statistical analysis

We used a generalized linear model (GLM) or multivariate regression model to analyze the relationships between multiple dependent variables (diagnostic tests results) and a single independent variable which was the absence or presence of PTB-associated histopathological lesions (focal, multifocal, and diffuse). Differences between these four groups in the last square means of the tests results were estimated by analysis of variance using the GLM procedure of the SAS software, Version 9.3, for Windows (SAS Institute Inc., Cary, NC). Correlations between different diagnostic tests were analyzed by principal component analysis (PCA) and the Pearson correlation test with the SAS9.3 software. The EBVs for milk yield (kg), milk fat (kg), milk protein (kg), longevity score, days open (DO), somatic cell score (SCS), functional merit index (ICOT), and the combined genetic index (ICO) were provided by the Spanish Federation of Holstein Cattle (CONAFE). The ICO is the Spanish official index for total genetic merit. It combines the different traits according to their economic importance and their genetic correlations. Correlations between the mean log_2_ copies of MAP DNA copies in feces and the EBVs for several traits were analyzed with the Spearman test correlation.

The discriminatory power of each diagnostic method to discriminate the presence or absence of focal and diffuse histopathological lesions was calculated individually for each test by Receiver Operator Characteristic (ROC) curve analysis using the *pROC* package from R 4.2.1. (34). The area under the curve (AUC) and optimal cut-off point to accurately classify samples were calculated for each test individually. Diagnostic methods with AUC values ≥0.9 were considered to have excellent discriminatory power, between 0.8 < AUC < 0.9 would indicate that the test has good discriminatory power, and 0.7 < AUC < 0.8 fair discriminatory power. The optimal cut-off values for sensitivity and specificity of each test were determined by using the Youden Index, which defines the optimal cut-off point as the value in which the sum of specificity and sensibility is the highest. Statistical analysis resulting in *P*-values lower than 0.05 were considered to be significant.

## Results

### Optimization of the ddPCR assay using DNA from MAP bacteriological culture

As with qPCR, optimizing the annealing temperature of the ddPCR assay is one of the most critical parameters. The optimum annealing temperature was determined by testing a range of temperatures above and below the calculated Tm of the primers (55, 57, 61, and 65°C). A total of 66 ng of MAP DNA isolated from a bacterial culture and 200 nM of primers were used in the assay. Restriction digestion of the DNA samples was performed directly in the ddPCR reaction as indicated in materials and methods. The negative template control (NTC) contained sterile water instead of DNA. As seen in Supplementary Figure 1A, the ddPCR could detect MAP in all the assessed temperatures. However, the annealing temperature of 65°C resulted in less non-specific amplification (rain) when compared with the other three temperatures. At 65°C, primer-dimers and off-target amplicons were not detected in the non-template control as low amplitude droplets. Hence, an annealing temperature of 65°C was used for further experiments.

To determine the best primer concentrations for the ddPCR assay, three different concentrations of F and R primers (200, 150, and 100 nM) were tested. A total of 5 ng of MAP DNA isolated from a bacterial culture were used in the assay. Restriction digestion of DNA samples (5 ng) was performed directly in the ddPCR reaction. Supplementary Figure 1B shows that the overall fluorescence amplitude of positive droplets increased with primer concentrations. Better separation between positive and negative droplets was observed when a 200 nM concentration of both primers was used. Thus, 200 nM primers concentration was used in further experiments. Using 66 ng of DNA (Supplementary Figure 1A), the target concentration was too high that every droplet contained DNA target and no negative droplets existed. Using only 5 ng of DNA (Supplementary Figure 1B), unspecific amplification (rain) was observed in the 1D plot. To test the effect of sample concentration, we performed a ddPCR assay using several concentrations of MAP DNA from a bacterial culture (Supplementary Figures 1C,D) and the optimum annealing temperature (65°C) and primers concentration (200 nM). Restriction digestion of DNA samples was performed directly in the ddPCR reaction. As seen in Supplementary Figure 1D, good band separation was observed at the three MAP DNA concentrations used. Since enough negative droplets are required to apply Poisson statistics and to calculate DNA concentration, 10 ng of DNA isolated from MAP culture was used as the positive control (201 negative droplets) in subsequent ddPCR assays. As seen in the tables below the Supplementary Figures 1C,D, no positive droplets were detected in the NTC.

### Optimization of ddPCR assays using DNA isolated from fecal samples

Several concentrations of DNA isolated from three different fecal samples with a MAP-positive qPCR result were tested by ddPCR (Supplementary Figures 2A–C). As seen in Supplementary Figure 2A, adding 205 ng of total DNA per 21 μl of reaction caused the positive and negative droplets to have poor separation and less droplet yield (< 10,000 total droplets). As seen in Supplementary Figures 2A,B, as the total added DNA concentration increased, the positive fluorescence amplitude decreased and the negative fluorescence amplitude increased. This problem was solved by performing a HindIII restriction digestion on the DNA before ddPCR. As seen in Supplementary Figure 2C, high concentrated samples without previous digestion produced only single amplitudes which do not allow distinction between positive and negative droplets (line 2). However, pre-digestion of the DNA sample (568 and 284 ng) with HindIII allowed to test larger amounts of total DNA and provided good droplet yield; 16,962 and 17,707 total droplets, respectively. These DNA concentrations corresponded to 9 and 4.5 μl of the HindIII restriction reactions. Since MAP DNA can be found in very low amounts in clinical samples, two ddPCR reactions containing 9 and 2 μl of HindIII-digested DNAs were used in subsequent ddPCR assays to ensure that one of them was within the optimal digital range. In the majority of the tested clinical samples, positive results were obtained with both reactions. When positive results were obtained with the high and low concentrations of amplified DNA template, non-statically significant differences in copies of MAP DNA/μl were observed (*P* = 0.29). As seen in the tables below the Supplementary Figure 1, no positive droplets were detected in the NTC. Although not included in Supplementary Figure 2, no positive droplets were detected when a DNA sample from a fecal sample negative to a PCR designed to amplify a fragment of the IS900 MAP sequence was included as a negative control.

### Quantification of MAP DNA in fecal samples by ddPCR and comparison with routine diagnostic tests

**Figure 1:**
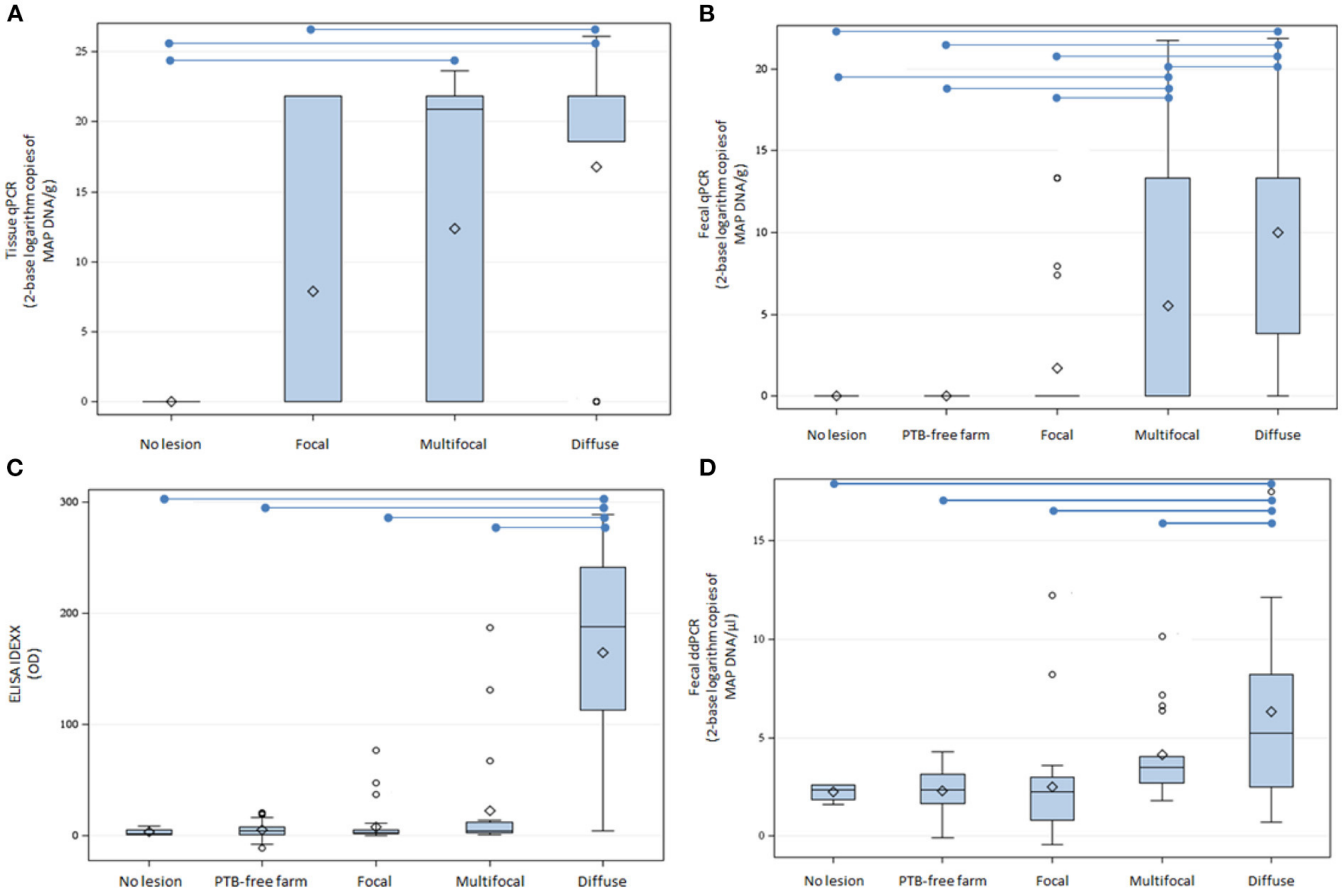
Tissue qPCR, fecal qPCR, ELISA, and fecal ddPCR results. **(A)** Distribution of tissue qPCR results (log_2_ copies of MAP DNA/gr). **(B)** Boxplot showing the log_2_ copies of MAP DNA/gr of feces obtained by fecal qPCR. **(C)** Distribution of the ELISA OD results. **(D)** Distribution of log_2_ copies of MAP DNA/μl estimated by fecal ddPCR. The plots represent the interquartile range and the whiskers represent the 95% range. Lines and diamonds within the boxes represent the median and the mean of each group, respectively. Blank dots represent outliers. Blue lines on the top of the figures represent statistically significant differences (*P* ≤ 0.05).

DNA was isolated from fecal samples from 69 cows from a PTB-free farm and 67 slaughtered cows without lesions (*N* = 4) or with focal (*N* = 29), multifocal (*N* = 20), and diffuse lesions (*N* = 14) in gut tissues and lymph nodes. Isolated DNA was digested with HindIII and 9 and 2 μl of the HindIII-digested DNAs were analyzed by fecal ddPCR. As seen in Figure 1, the mean log_2_ of copies of MAP DNA/μl estimated by fecal ddPCR (Figure 1D) showed a similar pattern to the fecal qPCR (Figure 1B), ELISA IDEXX (Figure 1C), and tissue qPCR (Figure 1A) results, with the highest values of the three tests corresponding to the group of animals with diffuse lesions. Fecal samples from cows with diffuse lesions had higher concentrations of MAP DNA (13,791.2 copies/μl) than samples from other cows (multifocal: 78.9 copies/μl, focal: 177.2 copies/μl, no lesion: 4.9 copies/μl, PTB-free farm: 5.6 copies/μl) (*P* ≤ 0.0001).

### Quantification of MAP DNA in whole-blood samples by ddPCR and comparison with the ABCA13 ELISA results

**Figure 2:**
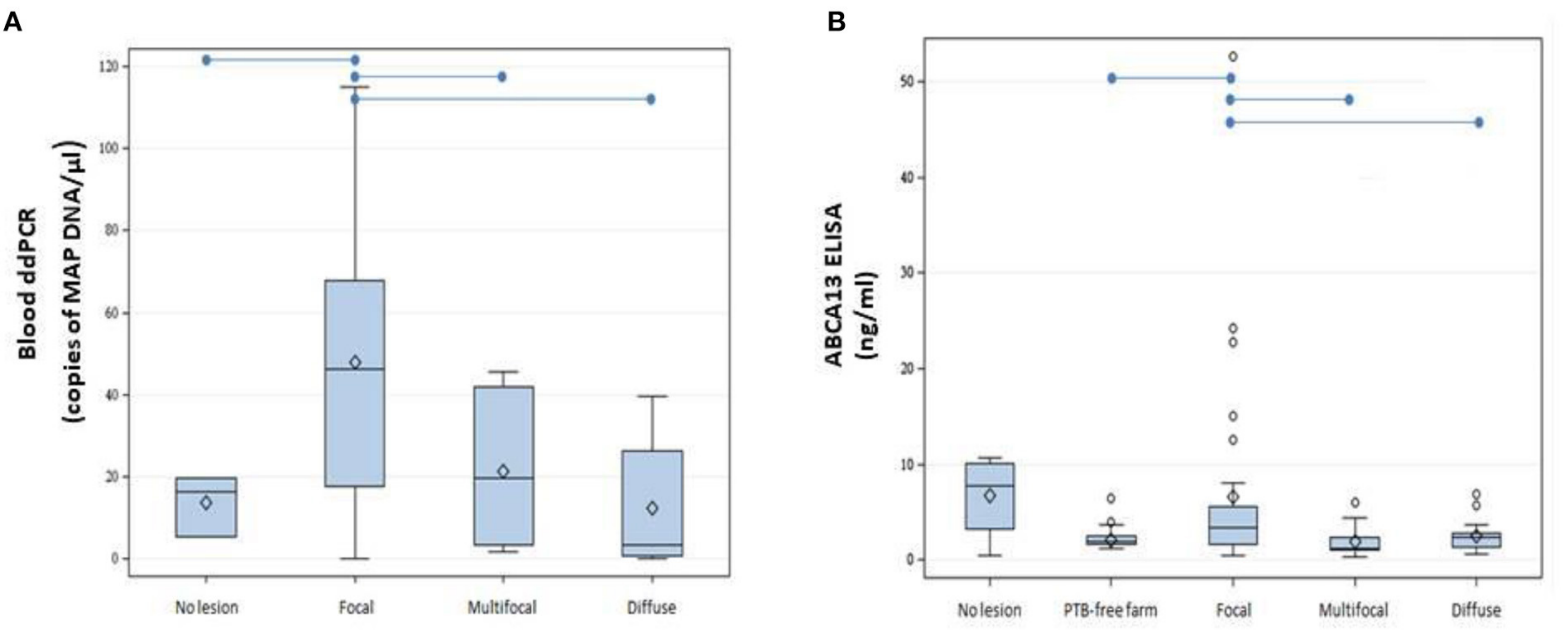
Boxplots showing the distribution of ABCA13 levels and copies of MAP DNA/μl obtained using blood ddPCR. **(A)** Distribution of copies of MAP DNA/μl obtained using blood ddPCR in cows without lesions and with different PTB-associated lesions. **(B)** Results of the ABCA13 levels measured by ELISA in serum samples of 61 cows from the PTB-free farm and from 74 slaughtered cows without lesions (*N* = 4) or with focal (*N* = 32), multifocal (*N* = 21), and diffuse lesions (*N* = 17) in gut tissues and lymph nodes. The plots represent the interquartile range and the whiskers represent the 95% range. Lines and diamonds within the boxes represent the median and the mean of each group, respectively. Blank dots represent outliers. Blue lines on the top of the figures represent statistically significant differences (*P* ≤ 0.05).

Blood samples were obtained from slaughtered cows without lesions (*N* = 3) or with focal (*N* = 14), multifocal (*N* = 13), and diffuse lesions (*N* = 8) in gut tissues and lymph nodes. Isolated DNA was digested with HindIII and 9 and 2 μl of the HindIII-digested DNAs were analyzed by ddPCR. As seen in Figure 2A, DNA isolated from the blood of cows with focal lesions had higher concentrations of MAP DNA (47.7 copies/μl) than samples isolated from cows without lesions (13.7 copies/μl) or with multifocal (18.1 copies/μl) and diffuse (12.4 copies/μl) PTB-associated lesions (*P* ≤ 0.05). The quantification of the ABCA13 transporter by ELISA showed similar results to the blood ddPCR, being the results of both tests higher in the animals with focal lesions when compared with the other groups (Figure 2B).

### Correlations between the results of the microbiological, immunological, and PCR test results

**Figure 3:**
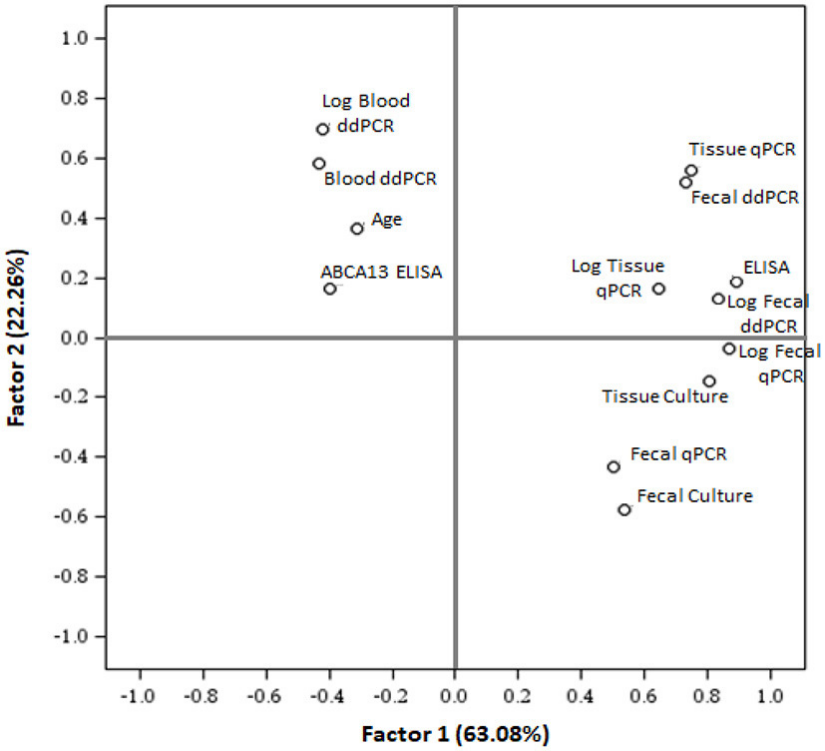
Principal component analysis to cluster the results of the test included in the study. PCA plot (PC1 vs. PC2) illustrates the distribution of all different tests results and the mean age of the animals included in the study. ELISA, ELISA for the detection of anti-MAP antibodies; ABCA13 ELISA, ELISA for the detection of the bovine ABCA13 transporter; Log, base-2 logarithm.

To cluster the results of all the diagnostic tests included in the study, a PCA was performed (Figure 3). The first two components (PC1 and PC2) explained 85.34% of the variance, and clustered together the results of blood ddPCR, ABCA13 ELISA, and age, which suggests that the animals with higher copies of MAP DNA in blood and ABCA13 levels have a longer lifespan. A second cluster included the results of the fecal ddPCR and qPCR, tissue qPCR, ELISA, and tissue and fecal culture. To analyze if the correlations between the independent variables included in the analysis were statistically significant, a Pearson correlation test between variables was performed and the results are presented in Table 1. As previously seen in the PCA, positive and statistically significant correlations (*r* > 0, *P* ≤ 0.05) were observed between the results of the fecal ddPCR (log_2_ copies of MAP DNA/μl) and the fecal and tissue qPCR, fecal and tissue culture, and ELISA for the detection of MAP antibodies. In contrast, blood ddPCR results (log_2_ copies of MAP DNA/μl) negatively correlated (*r* < 0, *P* ≤ 0.05) with the fecal qPCR, and with the fecal and tissue culture results. Although not statically significant, the positive correlation between the blood ddPCR and ABCA13 ELISA results and the negative correlation between the blood ddPCR and the ELISA for the detection of MAP antibodies suggests that blood ddPCR and the ABCA13 ELISA are more suitable for the detection of animals with low levels of MAP antibodies and MAP load in feces and tissues.

**Table 1:**
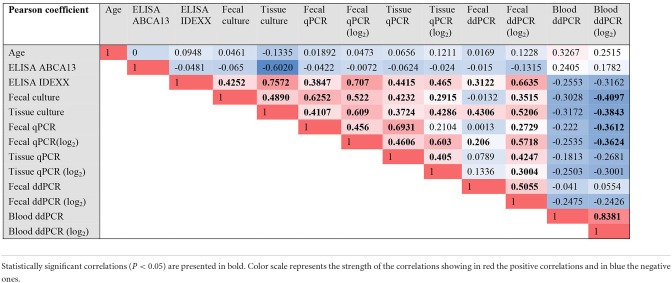
Pearson (*r*) correlation coefficients of the comparisons between all the results.

### Correlations between the results of the ddPCR and estimated breeding values for several traits

The EBVs for milk yield (kg), milk fat (kg), milk protein (kg), somatic cell score (SCS), longevity score, days open (DO), functional merit index (ICOT), and the combined genetic index (ICO) for all the animals included in the study were obtained from CONAFE. The ICO combines the different traits according to their economic importance and their genetic correlations. The SCS is the arithmetic mean of the somatic cells that is transformed using a base-2 logarithmic function (35). DO is the time from when a cow calves until it conceives. A positive correlation (ρ = +0.24, *P* = 0.042) between the fecal ddPCR results (log_2_ copies of MAP DNA/μl) and EBVs for the SCS was observed which suggests that animals with higher MAP load in feces estimated by ddPCR also have higher SCS and, therefore, are prone to develop PTB clinical signs and mastitis. In contrast, a negative correlation between the fecal ddPCR results (log_2_ copies of MAP DNA/μl) in feces and DO was observed (ρ = −0.40, *P* = 0.0006). No statistically significant correlations were observed between the blood ddPCR results and the EBVs of the traits included in the ICO.

### Diagnostic performance of all the quantitative methods included in the study

The AUC values, optimal cut-off values, specificities, and sensitivities of all the methods included in the study were estimated by ROC analysis (Table 2). For the detection of cows with diffuse lesions, the ELISA for the detection of MAP antibodies showed excellent discriminatory power (AUC = 0.95), and the fecal ddPCR (AUC = 0.80) and fecal qPCR (AUC = 0.88) showed good discriminatory power (0.8 < AUC < 0.9). For the detection of cows with focal lesions, most tests showed poor discriminatory power (AUC < 0.7), except for the blood ddPCR which showed fair discriminatory power (AUC = 0.78, sensitivity 71%, specificity 100%). However, its discriminatory power for the detection of animals with diffuse lesions was poor (AUC = 0.62).

**Table 2:** Diagnostic performance of the quantitative methods included in the study for the detection of animals with focal and diffuse PTB-associated lesions.

	**Fecal ddPCR (copies of MAP DNA/μl)**	**Blood ddPCR (copies of MAP DNA/μl)**	**ELISA ABCA13 (ng/ml)**	**ELISA IDEXX (OD)**	**Fecal qPCR (copies of MAP DNA/gr)**	**Tissue qPCR (copies of MAP DNA/gr)**
**No lesion vs. focal lesion**						
Cut-off	2.71	23.72	3.11	3.89	84.59	2
Sensitivity	0.48	0.71	0.53	0.68	0.15	0.46
Specificity	0.76	1	0.89	0.54	1	0.8
AUC	0.59	**0.78**	0.63	0.54	0.57	0.61
**No lesion vs. diffuse lesion**						
Cut-off	19.95	3.19	2.25	19.35	7	194,809.5
Sensitivity	0.64	0.5	0.58	0.88	0.76	0.76
Specificity	1	1	0.61	0.97	1	0.8
AUC	0.8	0.62	0.49	**0.95**	0.88	0.78

The highest AUC values are presented in bold.

## Discussion

ELISA, fecal qPCR, and fecal bacteriological culture are suitable for confirming clinical cases, but they have limited applicability in prevalence studies and eradication programs that require knowing the infectious status of all the animals in the herd. Since ddPCR can measure small amounts of nucleic acids with greater reproducibility than qPCR (36), we developed a ddPCR system for the detection and quantification of MAP in feces and blood collected from animals with distinct PTB-associated lesions. Several key performance parameters that can affect the separation of positive and negative droplets including primer concentration and annealing temperature were assessed. Our results showed that undigested DNA at high concentrations failed in packaging DNA into droplets, compromising the performance of the assay. Therefore, two concentrations of pre-digested DNA from clinical samples, high (9 μl) and low (2 μl), were analyzed to find the concentration that fell in the range of accurate quantification by ddPCR. The target concentrations were calculated based on the Poisson distribution and the individual data from the animals included in the study is presented in Supplementary Table 1. It should be noted that no positive droplets were detected in any of the NTC reactions during our study which ensures the reliability of the results. When positive results were obtained with the high and low concentrations of the DNA template, non-statically significant differences in copies of MAP DNA/μl were observed between the two DNA concentrations (*P* = 0.29).

Fecal ddPCR results showed that samples from cows with diffuse lesions yield significantly more copies of MAP DNA than samples from cows without or with less severe lesions (*P* < 0.0001), which suggests that this test could be useful to detect MAP in the clinical stages of the disease, similarly to fecal qPCR, ELISA, and bacteriological culture. Fecal ddPCR detected only a few positive droplets (mean = 4.9 copies/μl) in the group of cows with no lesion at slaughter and in the group of animals from the PTB-free farm (mean = 5.6 copies/μl). We cannot exclude the possibility that this signal, in some cases, represents a true positive animal, but the probability is low. These very low MAP DNA copy numbers/μl might represent the detection limit of the method, a marker of false positives. Fecal ddPCR (cut-off value = 19.95 copies of MAP DNA/μl) showed a sensibility of 64%, specificity = 100%, and AUC = 0.80 for the detection of cows with diffuse lesions.

A positive correlation (ρ = +0.24, *P* = 0.042) between the fecal ddPCR results (log_2_ copies of MAP DNA/μl) and the EBV for the SCS was observed. This correlation suggests that animals with higher MAP load in feces estimated by fecal ddPCR also have higher SCS EBV and, therefore, are at risk of developing mastitis. On the contrary, a negative correlation (ρ = −0.40, *P* = 0.0006) between the fecal ddPCR results (log_2_ copies of MAP DNA/μl) and DO (number of days from calving to conception) was also observed. Previously, Lombard et al. (37), found a decrease in DO for cows with a strong positive ELISA but no overall difference between positive and negative ELISA. Other studies have reported that the mean DO was not statistically significant between fecal culture positive and negative cows (38). It should be taken into account that differences in DO between test positive and negative cows are probably the result of earlier culling and breeding decisions, reflected in the statistical analysis results, as opposed to an effect of the test result. Tests positive cows that bred back promptly could be retained in the herd whereas those that did not were probably more likely to be culled.

Although the number of samples tested by blood ddPCR (*N* = 38) was lower than the number of tested fecal samples (*N* = 135), blood ddPCR results along with the ABCA13 transporter levels measured by ELISA were significantly higher in cows with focal lesions than in cows without or with more severe lesions and clustered together with the age of the animals. These results are particularly relevant, since they suggest that blood ddPCR could be used to diagnose cows with focal lesions and very low amounts of antibodies against MAP and MAP load in feces and gut tissues. Therefore, selecting the suitable sample type (blood or feces) to detect MAP is critical for the diagnosis of MAP-infected animals in different stages of the infection. For clinical PTB, suitable samples for qPCR and ddPCR would be feces but for subclinical PTB the suitable sample would be the peripheral blood. In our study, blood ddPCR was able to detect low levels of MAP DNA in subclinical animals with focal lesions. Unfortunately, blood qPCR could not be performed which would have led to a more complete comparison between ddPCR and qPCR assays. Further studies will focus on calculating the performance, optimal cut-off value, and cost-benefit of blood ddPCR using a larger number of animals with characterized lesions in gut tissues.

The principal component analysis showed that fecal ddPCR results clustered together with fecal qPCR, fecal culture, and ELISA for the detection of anti-MAP antibodies. In contrast, blood ddPCR clustered together with the ELISA ABCA13 results, which is particularly sensitive for the detection of subclinical animals with focal lesions (26, 27). Blood ddPCR (cut-off value = 23.72 copies of MAP DNA/μl) showed the highest sensibility (71%), specificity (100%), and AUC (AUC = 0.78) for the detection of subclinical cows with focal lesions. Our results showed a negative correlation between blood ddPCR and ELISA, and agree with previous studies (39). One interpretation is that each method detects different stages of MAP infection because their respective targets (bacteria and antibodies) do not have parallel dynamics. This explanation is consistent with the finding that ELISA is rarely positive in animals in the early phases of infection, possibly because MAP-loaded phagocytic cells would circulate from the intestinal lymphoid tissue to other locations very early after the infection. Haematogenous spread of infected macrophages out from Peyer's patches might result in the development of diffuse intestinal lesions in susceptible individuals (5). Similarly, studies on *Mycobacterium tuberculosis* have evidenced the potential of blood and plasma ddPCR to contribute to the early diagnosis of extrapulmonary tuberculosis (TB) in patients lacking respiratory symptoms and in infant TB patients, from whom it is impossible to obtain sputum samples (21, 22, 40–42). ddPCR and metagenomic next-generation sequencing (mNGS) have been recently compared for rapid and accurate detection of pathogens in patients with bloodstream infections (43). The ddPCR showed a higher detection rate of blood pathogens than the mNGS assay (88 positives in ddPCR vs. 53 positives in mNGS). Similarly, other studies have reported that some common pathogens detected by blood culture were missed by mNGS (44, 45), suggesting that plasma mNGS still needs improvement.

Although some studies have demonstrated that ddPCR is more resistant to inhibitors than qPCR due to sample partitioning (46, 47), ddPCR might remain susceptible to some inhibitors. For the application of ddPCR in PTB diagnosis, the inclusion of a positive internal control to confirm negative results is needed. When performing intercalating dye reactions in qPCR, the quantification of multiple amplification products from one reaction mixture is unachievable as only a single fluorescent measurement is made in each detection channel. However, with ddPCR, variations in the fluorescence signal intensity of EvaGreen due to the mass of DNA present in each droplet can be utilized for multiplexed detection in a single fluorescence channel (48). EvaGreen-based multiplexing can be achieved by varying amplicons length, primers concentration or optimal annealing temperature to generate different levels of amplified DNA mass/positive droplet fluorescence for each target sequence. As result, multiple positive droplet clusters can be observed and assigned to each target of interest for quantification in a single well. Further experiments will focus on the inclusion of an internal positive control in the EvaGreen-based ddPCR developed in the current study.

Despite the many advantages offered by the ddPCR, some disadvantages of this technology include that the ddPCR is more time-consuming than qPCR, the reaction volumes are limited, and the chances of contamination are higher. Whether ddPCR and qPCR positive results represent dead or alive MAP requires verification by other methods. Excluding the initial cost of acquiring the instruments, the cost of the analysis is less for ddPCR (QX200; 3.16 € per sample) than for qPCR (10.80 € per sample) (24). In addition, ddPCR assays do not require the preparation of reference standards and the construction of a standard curve. All of the mentioned advantages explain why ddPCR is gaining attention when it comes to the diagnosis of pathogens in the early stages of chronic infections. The goals of PTB programs vary from eradication in regions/countries of low prevalence to control in regions/countries with high prevalence. Therefore, the potential application of the fecal and/or blood ddPCRs developed in the current study will depend on MAP prevalence and the cost-benefit that each farmer can assume. As PTB has a long incubation period before the disease becomes evident, the early diagnosis of subclinical animals is information of great value for the farmer. Using this information, the farmer could decide on an adequate protocol that could help control or eradicate PTB, ultimately reducing the prevalence of this widespread disease.

## Conclusions

In conclusion, a F57-targeted ddPCR was shown to be an accurate assay for the quantification of MAP in feces and blood. The results showed that the ddPCR can detect low levels of MAP DNA and the potential to be used to diagnose clinical and subclinical PTB using fecal and blood samples, respectively. However, a multicenter study with a larger number of samples is required to confirm these results.

## Data availability statement

The datasets presented in this study can be found in online repositories. The names of the repository/repositories and accession number(s) can be found in the article/Supplementary material.

## Ethics statement

The animal study was reviewed and approved by Animal Ethics Committee of the Servicio Regional de Investigación y Desarrollo Agroalimentario (SERIDA) and authorized by the Consejería de Agroganadería y Recursos Autóctonos of the Principality of Asturias (approval code PROAE 29/2015 and PROAE 6672019).

## Author contributions

GBB and MC performed ddPCR optimization and ddPCR assays from MAP culture, feces, and blood. RC, CBV, and NI performed the ELISAs and bacteriological culture from feces and tissues. RC and JA performed DNA extraction and qPCR from fecal samples. MB helped with DNA isolation from bacteriological culture. AG provided technical assistance with ddPCR and data analysis. RJ and GBB performed the statistical analysis of the data. GBB and MAH wrote the manuscript. GBB generated the figures and tables. MAH and RC contributed to the study design, data acquisition, and data analysis. MAH conceived and coordinated the project. All authors revised and approved the final version of the manuscript.

## Funding

Financial support for this study was provided by a grant (RTI2018-094192-R-CO1) funded by Spanish Ministry of Science and Innovation (MCIN/AEI/10.13039/501100011033) and by European regional Funds (FEDER, Una manera de hacer Europa). MC, GBB, and CBV have been awarded fellowships from MCIN/AEI/10.13039/501100011033 and FSE Invierte en tu futuro; grants FPI2016-00041, PRE2019-090562 and CPD2016-0142, respectively.

## Conflict of interest

The authors declare that the research was conducted in the absence of any commercial or financial relationships that could be construed as a potential conflict of interest.

## Publisher's note

All claims expressed in this article are solely those of the authors and do not necessarily represent those of their affiliated organizations, or those of the publisher, the editors and the reviewers. Any product that may be evaluated in this article, or claim that may be made by its manufacturer, is not guaranteed or endorsed by the publisher.
