# Supplementary material for: Evaluation of a droplet digital PCR assay for quantification of Mycobacterium avium subsp. paratuberculosis DNA in whole-blood and fecal samples from MAP-infected Holstein cattle
Source: Front Vet Sci. 2022 Sep 30;9:944189. doi: 10.3389/fvets.2022.944189 (PMC9563315; doi:10.3389/fvets.2022.944189)

**Supplementary Figure 1. Optimization of the ddPCR assay using MAP DNA from a bacteriological culture. A.** The gradient of annealing temperatures (65 ˚C, 61.2 ˚C, 57.1 ˚C, 55 ˚C) is represented in a 1D plot with positive (blue) and negative (gray) droplets. Each temperature was run in duplicate. **B.** 1D plot of the optimal primer concentration assay (200 nM, 150 nM, 100 nM). **C.** 1D plot showing descending concentrations of MAP DNA (25 ng, 10 ng, 5 ng, and 1 ng) in duplicate. **D.** 1D plot showing descending concentrations of MAP DNA (15 ng, 12.5 ng, and 10 ng). 1D plots show droplets (event number) versus fluorescence amplitude. The tables below the figures show the number of total and positive droplets and the copies/µl of the F57 sequence. In all the ddPCR assays, restriction digestion of DNA samples was performed directly in the ddPCR reaction. The negative template control (NTC) contained sterile water instead of DNA. QuantaSoft software will return “No Call” for wells with too many positive droplets to apply Poisson statistics.


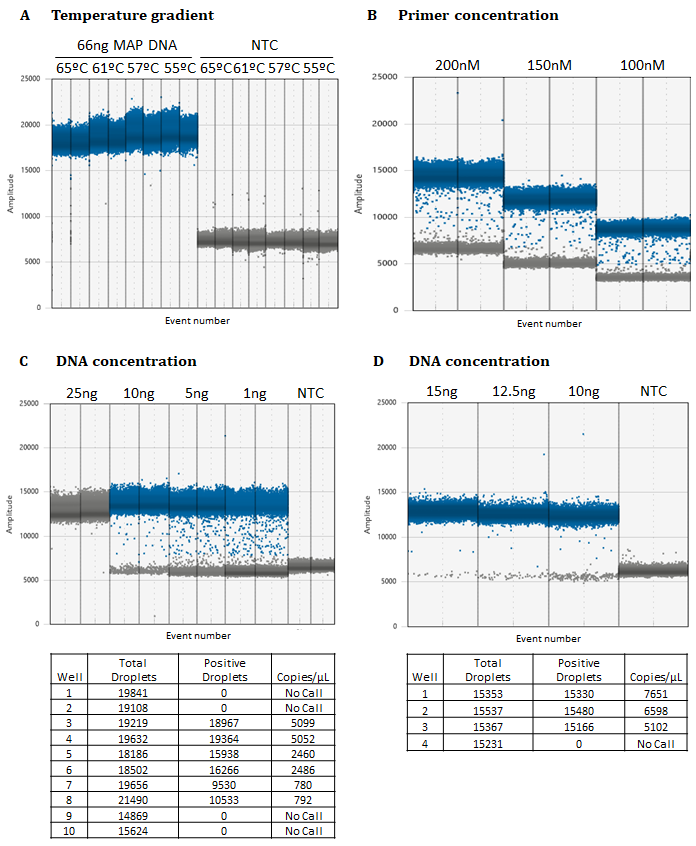

Supplement: Supplementary file 2 [file Data_Sheet_1.docx]
