# Supplementary material for: Evaluation of a droplet digital PCR assay for quantification of Mycobacterium avium subsp. paratuberculosis DNA in whole-blood and fecal samples from MAP-infected Holstein cattle
Source: Front Vet Sci. 2022 Sep 30;9:944189. doi: 10.3389/fvets.2022.944189 (PMC9563315; doi:10.3389/fvets.2022.944189)

**Supplementary Figure 2. Optimization of ddPCR assays using DNA isolated from fecal samples. A.** 1D plot of a ddPCR assay using an increasing concentration of DNA isolated from a positive PCR fecal sample (25, 66, and 205 ng). Each concentration was run in duplicate. **B.** 1D plot of a ddPCR assay using an increasing concentration of DNA isolated from a positive PCR fecal sample (80, 100, 120, 140, 160, 180 ng). In **A** and **B,** restriction digestion of DNA samples was performed directly in the ddPCR reaction. **C.** Effect of pre-PCR digestion of the DNA samples on ddPCR results. Line 1, No template control (NTC); line 2: 758 ng of undigested DNA from a positive PCR fecal sample; lines 3 and 4: 568 and 284 ng of digested DNA from a positive PCR fecal sample, respectively. In the tables, the number of total and positive droplets and MAP DNA copies/µl are shown. The NTC contained sterile water instead of DNA. The positive control contained 10 ng of MAP DNA isolated from bacterial culture. *QuantaSoft* software will return a No Call for wells with too many positive droplets to apply Poisson statistics. The optimum annealing temperature (65 ˚C) and 200 nM primers concentrations were used in A, B and C. 1D plots show droplets (event number) versus fluorescence amplitude. Positive and negative droplets are presented in blue and grey, respectively.


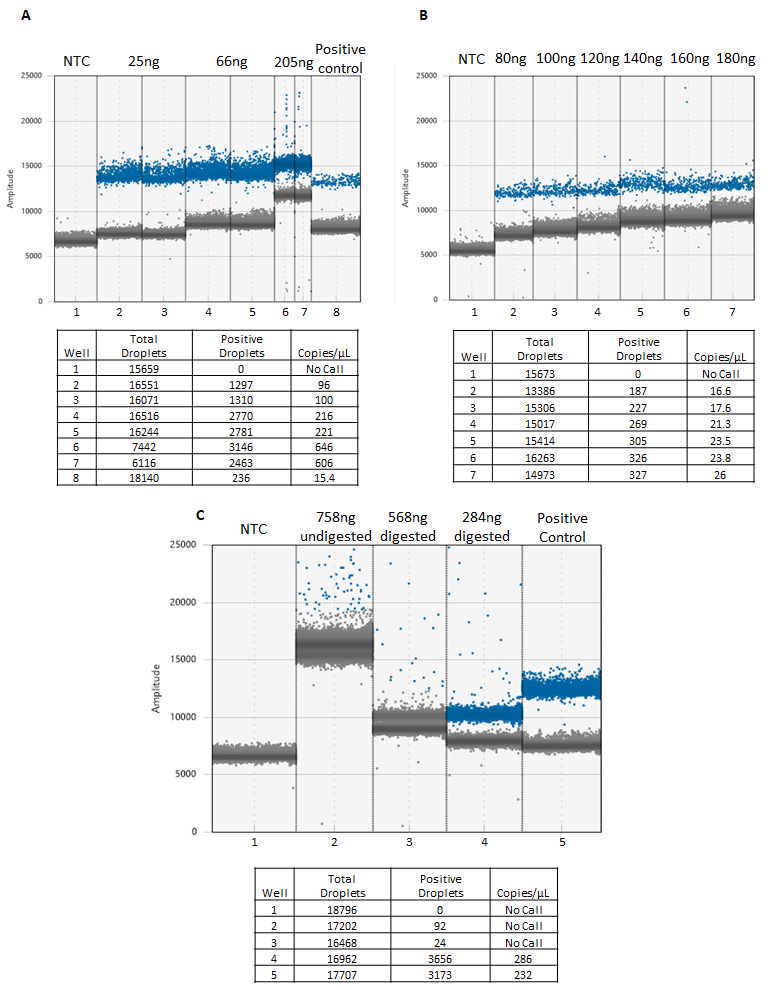

Supplement: Supplementary file 3 [file Data_Sheet_2.docx]
